# Packing for the journey: translating practice development into the digital realm

**DOI:** 10.3389/frhs.2026.1797479

**Published:** 2026-07-08

**Authors:** Katharina Gabl, Hanna Mayer, Theresa Clement

**Affiliations:** Department of Nursing Science with Focus on Person-centred Care Research, Faculty of Health Sciences, Karl Landsteiner University, Krems, Austria

**Keywords:** counselling, digital health, eHealth, facilitation, family caregivers, person-centred care, person-centredness, practice development

## Abstract

**Background:**

Digital technologies offer new opportunities to support family caregivers, a growing priority in ageing societies. Alles Clara is an Austrian digital service that provides low-threshold, individual support by professional counsellors from the fields of nursing and psychology. Central to its effectiveness is a person-centred approach to counselling. Moving beyond the conceptual debate on whether and how person-centred approaches align with digital services, we embarked on a transformational journey, translating practice development (PD) into the digital realm.

**Methods:**

Our journey unfolded in three phases: (1) Setting the Scene—analysing the app-usage and counselling characteristics through in-app tracking and user feedback; (2) Encompassing Change—co-creating a concept for PD in Alles Clara through a participatory approach together with counsellors (*n* = 10) and a newly-introduced practice facilitator; and (3) Packing for the Journey –outlining and structuring key PD strategies to sustainably enable and empower counsellors in providing person-centred counselling in Alles Clara.

**Results:**

Baseline data from 1,263 family caregivers and 342 counselling sessions highlighted the heterogeneity of users and their needs. Requests ranged widely, reflecting the diverse demands placed on digital counselling. Defining our destination meant exploring counsellors' shared values and vision. Identifying key concepts for PD in Alles Clara led us to take first steps to bring the concept into action. Drawing on counsellors' experiences and perspectives, we shaped our approach to PD and jointly drew the map for the path towards high-quality person-centred online counselling. The adapted PD framework provided a coherent structure for organizing developmental strategies and measures, translating abstract values into tangible, actionable practices within the digital environment.

**Conclusion:**

For PD to succeed in digital healthcare, maintaining human connection must remain a deliberate and continuous effort. The cue does not lie in choosing between technology and humanity, but in learning how to travel both. In this sense, packing for the journey ahead becomes a necessity: equipping ourselves with the principles, processes, and attitudes that preserve the human core of care, even as digital transformation continues to reshape practice. PD offers a compass for navigating this terrain, ensuring that the person remains at the very centre of care.

## 1 Introduction

Over the past two decades, digital technologies have transformed healthcare, driving the rapid expansion of eHealth services worldwide (1, 2). Digitalisation in healthcare has emerged as a subject of intense debate. On one hand, digital tools expand access, efficiency, and flexibility. On the other, there are pressing concerns about the dehumanization of care, the erosion of presence, and the risk of reducing relationships to transactions. The challenge, then, is not whether to digitalize care but how to do so without losing its human core.

This challenge is at the heart of Alles Clara, an Austrian digital counselling service designed to support family caregivers, the often invisible backbone of care provision (3, 4). In Austria, approximately 950,000 individuals provide care to relatives or loved ones (5). While caregiving plays a vital role in sustaining healthcare systems, it often entails profound physical, emotional, and social burdens (6). Addressing caregivers' needs has thus become both a national and global priority (7, 8). In the Alles Clara app, a data-secure messenger tool, family caregivers connect with professional counsellors from the fields of psychology and nursing, receiving free, flexible, and low-threshold yet personalized support. The primary mode of communication is asynchronous chat, with additional options for telephone and video counselling. Counsellors—nurses, psychotherapists, and psychologists—engage in online counselling as a flexible and complementary part of their professional practice (3).

A realist informed evaluation of Alles Clara uncovered a field of tension within two seemingly contradictory phenomena, and their joint occurrence being the intervention's unique feature: Family caregivers are both seen and not seen at the same time. Flexibility and low-threshold access are key features; anonymity plays an important role. Yet, the key to successful counselling lies in the establishment of a trusting relationship, placing the caregiver, as a person, at the centre. Rather than an additional feature, person-centredness is the central mechanism, the crucial aspect driving Alles Clara's impact [(3); Gabl et al., under review; (9)]. While person-centredness is already an established practice, evolving needs and the specific conditions of digital counselling call for deliberate efforts to uphold, deepen, and sustain this foundational orientation. Building on these insights, we move beyond the conceptual debate on whether and how person-centred approaches align with digital services. Instead, we focus on the process of fostering person-centredness within the new and challenging context of the digital and often anonymous realm—through Practice Development (PD).

This brings us to largely uncharted territory. PD, originally conceptualized within nursing, aims to achieve person-centred and evidence-based care, manifested through human flourishing and a workplace culture of effectiveness across healthcare settings (10). It is concerned with achieving increased effectiveness in providing care that is centred on persons' [original: patient] needs [(11), p.15], thereby enhancing quality in care. PD has a long-standing association with the development of person-centred cultures, practices, and ways of working and has proven effective in cultivating values-based practice in clinical and organizational environments (12). However, the systematic application of practice development approaches in digital contexts remains underexplored. Alles Clara thus provides a unique testing ground to explore how PD can be adapted to an online, anonymous, and asynchronous environment, where traditional cues of presence, authenticity, and connection are profoundly altered. The project presented in this paper takes on precisely this challenge. To support counsellors in fostering and sustaining person-centredness within the digital environment of Alles Clara, we embarked on a transformational journey, translating PD into the digital realm.

## 2 Materials and methods

### 2.1 Research design

Applying PD to a highly specific context like Alles Clara required careful adaptation to the digital and asynchronous nature of the service. Garbett and McCormack's (13) seminal work around PD, both the ontological underpinning in a transformative and emancipatory paradigm and the conceptual framework for PD, guided the structured implementation of PD within Alles Clara. In line with McCormack and Dewing's (14) conceptualization of PD as a “transformational journey”, the project unfolded in three overlapping phases: (1) setting the scene by assessing the usage of Alles Clara, (2) encompassing change by collaboratively developing an initial concept for PD, and (3) packing for the journey by outlining and structuring key strategies to sustainably enable and empower counsellors in providing high-quality person-centred counselling.

### 2.2 Research process

#### 2.2.1 Phase 1: monitoring

Continuous monitoring served to map the landscape of Alles Clara. Data collection and analysis was informed by insights around Alles Clara's impact derived from the previous Realist Evaluation [(3); Gabl et al., under review; (9)], forming the foundation for Phase 2: PD in Alles Clara.

##### 2.2.1.1 Data collection

Alles Clara provided us with anonymized data on user engagement and counselling dynamics from July 2022 to November 2024. Two primary data sources were used: tracking data from the Alles Clara app and questionnaire surveys completed by both family caregivers and counsellors after counselling sessions. Tracking data provided insights on user demographics, usage patterns, and counselling characteristics, while counsellor questionnaires captured qualitative aspects such as research time, referrals, and communication channels. This phase addressed three key questions: (1) Who are the users of Alles Clara? (2) How are counselling sessions structured? (3) How do caregivers and counsellors evaluate their experiences? These insights informed us of the next steps towards PD.

##### 2.2.1.2 Participants and recruitment

Family caregivers using Alles Clara and counsellors working within the service provided insights into their experiences through feedback opportunities integrated into the Alles Clara app. A notice within the app informed users about the potential scientific evaluation of their data. Participation was voluntary, and data collection adhered to ethical guidelines.

##### 2.2.1.3 Data analysis

Baseline survey and tracking data were analysed descriptively using MS Excel® and presented in tabular format.

#### 2.2.2 Phase 2: Co-creation

**Figure 1:**
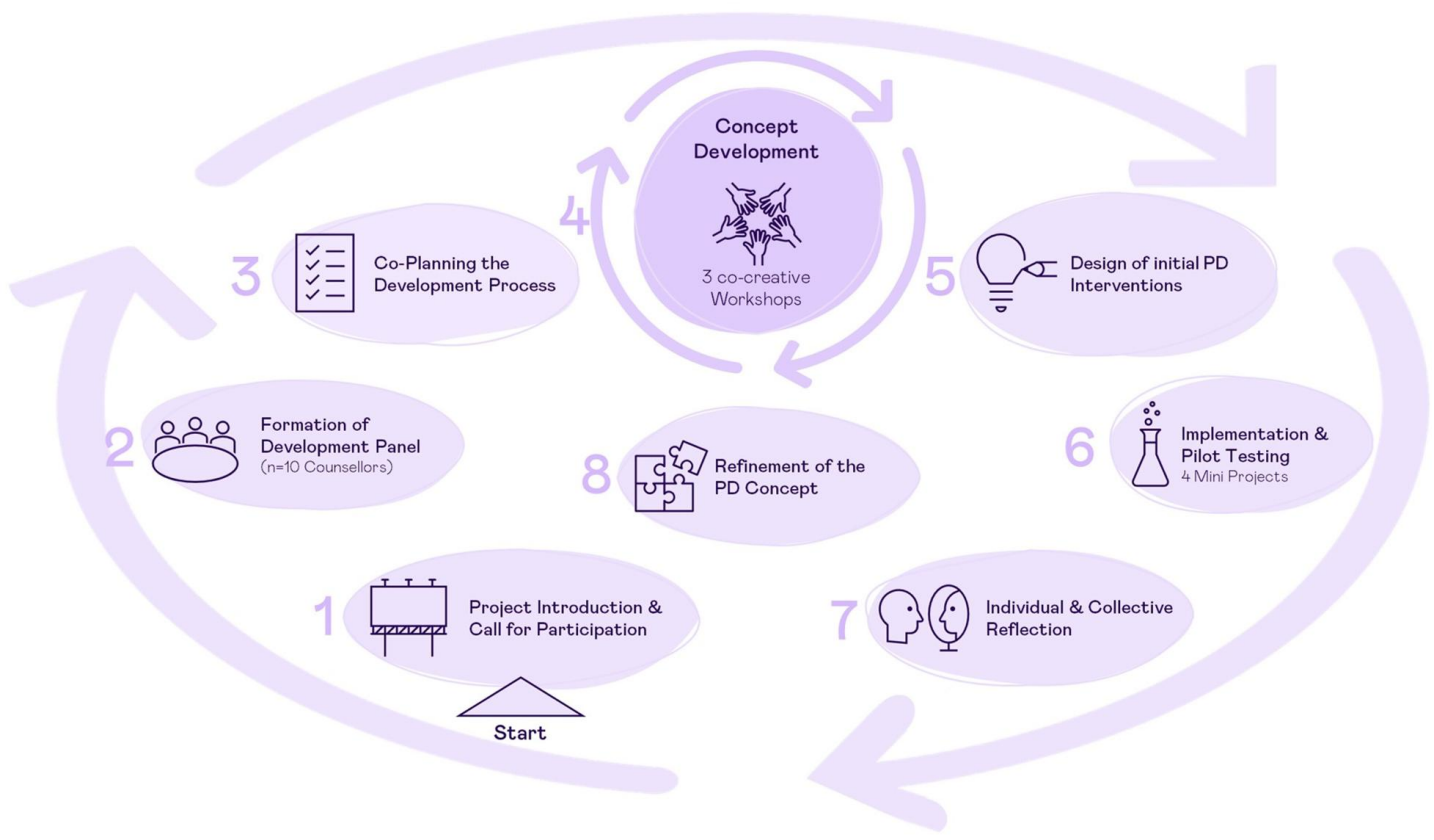
Phase 2: Co-creation.

While the quantitative baseline data from Phase 1 served as an initial situational analysis and provided an overview of counsellors' working condition, our PD journey commenced with charting new routes for transformation. To develop a concept for PD in Alles Clara we embraced a participatory approach, ensuring active practitioner involvement through a development panel of ten Alles Clara counsellors (C). The development process itself was structured along a creative learning cycle (see Figure 1). In three collaborative workshops (WS), the research team and panel co-created an initial concept for PD. In small-scale pilot projects PD instruments were tested for implementation. Phase 2 was supported by a dedicated facilitator who played a key role in guiding and embedding the process.

##### 2.2.2.1 Co-creation activities

Concept development in Workshops:

The development panel participated in three creative online workshops (via Zoom®, each scheduled for two hours), facilitated by the research team. The first workshop, “Defining Quality”, aimed to establish a shared understanding of high-quality counselling in Alles Clara. Participants identified and reflected on person-centred moments (15) and developed advertising slogans to capture essential aspects of counselling. The second workshop, “Idea Lab”, focused on ideating initial PD interventions. We drew on creative techniques, including role-based ideation [Disney Method by (16)], to foster an open-thinking process. The final workshop, “From Idea to Implementation”, refined the PD concept and planned the pilot testing of initial PD interventions within a series of mini-projects. Workshop discussions were audio recorded and transcribed for analysis.

Establishing Facilitation:

Facilitation plays a key role in empowering individuals and teams in PD (17, 18). To strengthen this process, we introduced a dedicated facilitator to support reflection and foster team development. The facilitator was introduced to their role through engagement with foundational PD literature, expert consultation, and structured self-reflection. In the course of the mini projects the facilitator conducted an initial session to test the format in the digital context. A final narrative interview with the facilitator focused on reflection of the implementation process and personal reflection on her role within the process. It was conducted via MS Teams®, audio recorded and transcribed for analysis.

Mini-Projects:

The development panel tested four initial PD interventions that were aligned closely with the newly developed PD concept. These included reflective tools, structured peer feedback formats and practice-oriented resources designed to support person-centred counselling. The implementation and pilot testing of these mini-projects took place from July to October 2024 and were led by two counsellors each. The facilitator supported the implementation and process reflection, while the research team assisted through providing structural support. Activities and experiences were documented using structured protocols and reviewed in a final workshop, which was also audio-recorded and transcribed for analysis.

##### 2.2.2.2 Participants and involvement

The development panel consisted of Alles Clara counsellors and was established to ensure active participation and continuous alignment with practice requirements. During an internal Alles Clara training session, all counsellors were informed about the project by the first and last author. Interested participants contacted the research team for further information. Participants were required to have been employed for at least four weeks. There were no exclusion criteria. Participation was voluntary, with informed consent obtained before involvement, and withdrawal was possible at any time without consequences.

The facilitation role is defined through four core competencies (17): (1) expertise in navigating contextual conditions, (2) understanding organizational levels and power dynamics, (3) effective communication skills, and (4) the ability to engage stakeholders. Within Alles Clara, effective facilitation required a deep understanding of the service's values, structures, and complexities, alongside a strong connection to the counsellors' role. The designated facilitator met these requirements as she had formerly supported the Alles Clara team as a counsellor herself and holding an occupational background in psychology and supervision. While playing a key role in supporting the co-creation process, the facilitator was not involved in data analysis and had no decision-making authority regarding research outcomes.

The research team combined expertise in person-centredness and PD, digital counselling, and realist evaluation. All authors were involved in the preceding evaluation of Alles Clara [(3); Gabl et al, under review; (9)], which informed contextual understanding while necessitating reflexive awareness during analysis. Familiarity with most of the counsellors and the facilitator from the previous project fostered a trusting relationship, which was a prerequisite for effective collaboration. Given the research team's involvement with Alles Clara and commitment to person-centred practice, reflexivity was integrated throughout the research process. Regular reflexive discussions were conducted within the research team to critically examine assumptions, interpretations, and analytical decisions.

##### 2.2.2.3 Data analysis

The research team conducted a content analysis [following the approach described by (19)] of the collected data for the PD concept. Audio recordings from the workshop sessions and the facilitation interview were transcribed verbatim. Activities and experiences from mini projects were already documented by the members of the development panel in text format. Transcripts and texts were read and reread to achieve familiarity with the data. The analysis progressed inductively from identifying meaning units to generating initial codes. Two members of the research team (anonymized for peer review) then organized these codes into categories and higher-order themes.

Preliminary findings were shared with the development panel participants before each subsequent workshop, allowing them to review and reflect on the results in advance. At the beginning of the workshops, these preliminary results were discussed among peers, and interpretations were adjusted as needed based on collective insights. To ensure alignment with established PD frameworks, the final phase of the project involved synthesizing the findings and integrating them into the PD model by Garbett and McCormack (13). This iterative approach facilitated a participatory refinement process, ensuring that the newly developed concept for PD in Alles Clara remained grounded in both empirical data and practitioner experience. Final presentation of the results includes categories supported by illustrative quotations from counsellors to enhance transparency and aid interpretation of the findings.

#### 2.2.3 Phase 3: synthesis

Our overarching goal was to support counsellors in fostering and sustaining person-centredness within the digital environment of Alles Clara. To achieve this, the PD model co-created in Phase 2 needed to be operationalized to be applicable and sustainable in everyday practice. The aim of Phase 3 was to translate the concept into a structure for organizing measures related to person-centredness and PD, enabling targeted development in Alles Clara. This comprised the synthesis of five data sources: the configurational model from the realist evaluation; the co-created PD concept; qualitative feedback from mini-projects and facilitator reflections; relevant literature on digital counselling and PD; and contextual insights from ongoing collaboration with the Alles Clara team.

The configurational model and the PD concept provided the primary theoretical orientation. From these frameworks, key concepts were derived as analytical lenses: “knowledge”, “time”, and “attitude” (from the configurational model), as well as “authentic engagement” and “facilitated active learning” (from the PD framework). The remaining sources (feedback from mini-projects and facilitator reflections, relevant literature on digital counselling and PD, and contextual insights from ongoing collaboration with the Alles Clara team) were reviewed alongside these frameworks to identify, refine, and contextualize practical measures that could operationalize the concepts within the Alles Clara setting.

Many measures supporting counsellors were already embedded in Alles Clara without being explicitly framed as PD strategies or systematically linked to these theoretical concepts. An initial exploratory analytical step was required to identify the underlying aims of these measures and the mechanisms they intended to address in relation to counsellors' practice and development. The identified measures were then deductively mapped onto the predefined conceptual categories. The categories themselves were further specified and translated into the concrete context of Alles Clara. The analysis followed an iterative logic, moving back and forth between aim identification and deductive clustering, allowing empirical insights from practice and theoretical concepts to continuously inform and refine each other throughout the synthesis process. The outcome is a set of strategies that represent a flexible and context-sensitive framework that can evolve alongside changing practice requirements and service conditions.

## 3 Results

Our findings are presented in three chapters. The journey began with mapping out the landscape we are navigating—the context of Alles Clara. We set the scene by outlining key insights into our users' demographics and the counselling practice. Next, we introduce the adapted PD concept for Alles Clara, which served as our compass on the path to person-centred digital counselling. Finally, we took stock of the tools and measures we have gathered along the way—practical approaches that have supported us so far and will continue to be essential. We present eight key strategies for PD in Alles Clara to ensure and sustain a thriving, person-centred culture in the digital realm.

### 3.1 Before we take off: A first glimpse of our point of departure

Taking a first glimpse at our starting point and providing some contextualization for the journey ahead, data from 1,263 family caregivers and 342 counselling sessions were included in the baseline analysis. Drawing on the presentation provided by Gabl et al. (under review), Table 1 contains basic user characteristics. Alles Clara users come from all over Austria and represent various age groups. The age group of 54–59 years has the highest representation at 23% (*n* = 296). 67% (*n* = 848) of users identify as female. About one-fifth (22%, *n* = 273) of registered users have already made use of counselling in Alles Clara at least once. The majority of counselling sessions remained active for more than two months (42%, *n* = 127). Most sessions (95%, *n* = 257) were conducted entirely via asynchronous chat, while an additional 4% (*n* = 13) included telephone counselling.

**Table 1:** User characteristics.

FAMILY CAREGIVERS	
Source	*Tracking Data N* *=* *1,263 [100%]*
SOCIODEMOGRAPHIC CHARACTERISTICS	
Gender	Identify as female: 67,14% (*n* = 848)
	Identify as male: 32,22% (*n* = 407)
	Identify as diverse: 0,63% (*n* = 8)
Age, years	18–23: 2,86% (*n* = 36)
	24–29: 6,44% (*n* = 81)
	30–35: 8,43% (*n* = 106)
	36–41: 12,57% (*n* = 158)
	42–47: 15,27% (*n* = 192)
	48–53: 19,57% (*n* = 246)
	54–59: 23,55% (*n* = 296)
	60–65: 8,19% (*n* = 103)
	66–71: 1,51% (*n* = 19)
	Older than 72: 1,59% (*n* = 20)

COUNSELLORS AT ALLES CLARA	
Source	*Tracking Data N* *=* *24 [100%]*
SOCIODEMOGRAPHIC CHARACTERISTICS	
Gender	Identify as female: 87,5% (*n* = 21)
	Identify as male: 12,5% (*n* = 3)
Profession-related characteristics	
Profession	Nurse: 87,5% (*n* = 21)
	Psychologist: 8,3% (*n* = 2)
	Psychotherapist: 4,2% (*n* = 1)

CONSULTATIONS	
Source	Tracking Data (*N* = 342)
Counselling status	Active: 11,70% (*n* = 40)
	Closure requested: 1,46% (*n* = 5)
	Concluded: 86,84% (*n* = 297)
Duration	< 1 day: 3,64% (*n* = 11)
	1 day—1 week: 7,95% (*n* = 24)
	1–2 weeks: 10,93% (*n* = 33)
	2 weeks—1 month: 26,82% (*n* = 81)
	1–2 months 27,81% (*n* = 84)
	> 2 months: 22,84% (*n* = 69)

Counsellors supported family caregivers in 47% of cases (*n* = 122) through knowledge transfer, in 8% (*n* = 22) through emotional support. In 36% of counselling sessions (*n* = 94), both knowledge transfer and emotional support were provided. The most frequently discussed topics included information on available support services (*n* = 168, 28.2%) and coping with emotional and psychological stress (*n* = 90, 15.1%). Other common topics included managing their daily life (*n* = 59, 9.9%) and financial matters (*n* = 59, 9.9%). In 37% (*n* = 91) of counselling sessions, family caregivers were referred to specific analogue services. Counsellors felt they were able to support family caregivers rather to very well in 92% of counselling sessions (*n* = 249).

Findings indicate an overall positive perception of counselling in Alles Clara among family caregivers. In 66% of cases (*n* = 70), family caregivers rated the answers to their questions as very good, while 23% (*n* = 24) rated them as rather good. Regarding response time, 72% (*n* = 76) were very satisfied, 22% (*n* = 23) were rather satisfied. 67% of family caregivers (*n* = 62) reported feeling very good about chatting or talking on the phone with their counsellors, 23% (*n* = 21) felt rather good. Overall, 71% (*n* = 75) of family caregivers rated counselling as very good, 22% (*n* = 23) as rather good. 48 respondents (42%) strongly agreed with the statement “I feel well taken care of at Alles Clara” with the highest score (10 points). 39 people (34%) chose answers in the upper range (7–9) of the scale.

### 3.2 The compass of change: A concept for practice development in alles Clara

**Figure 2:**
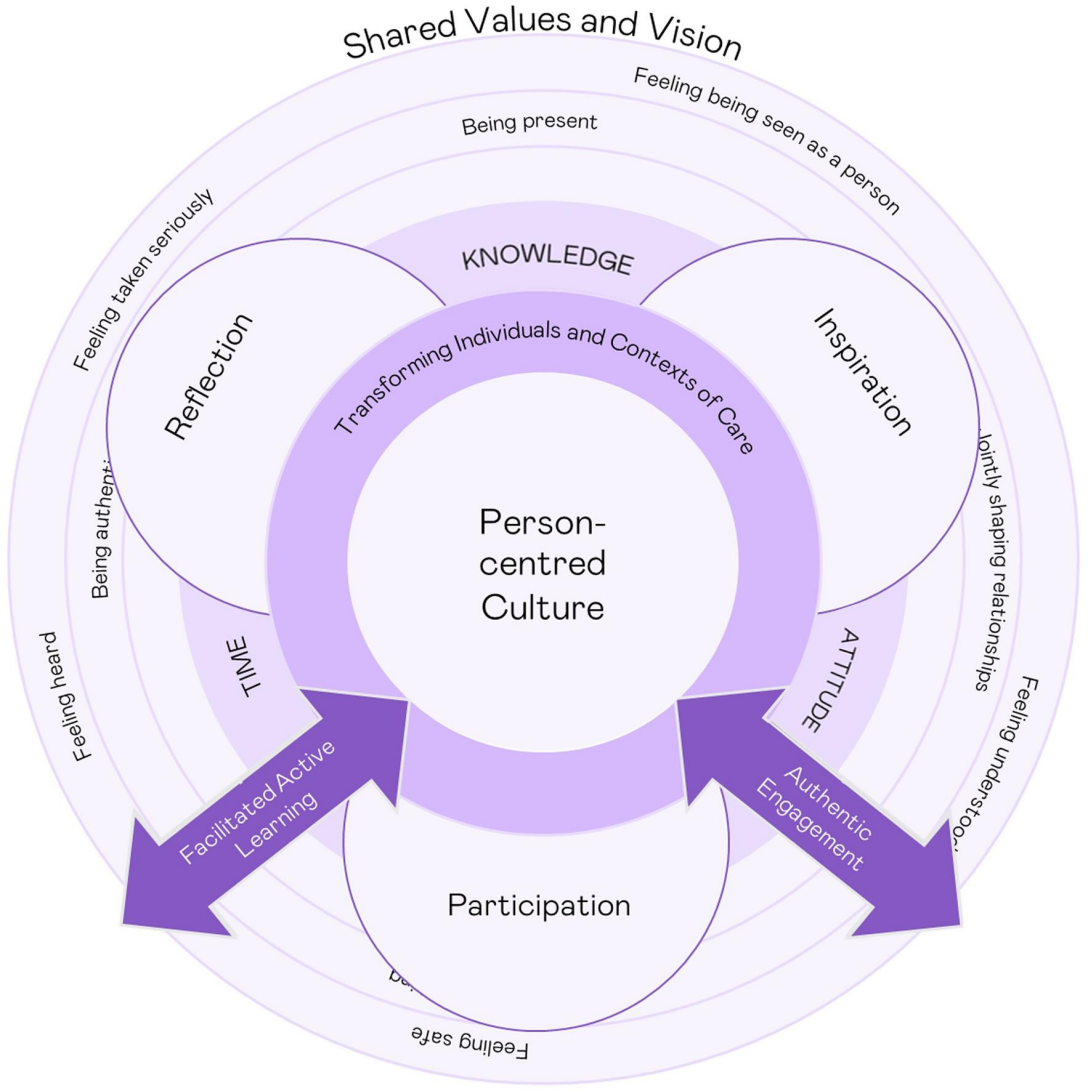
Concept for Practice Development in Alles Clara [adapted from (12)].

Building on the insights gained from the situational analysis of counselling practice and counsellors' working conditions, we moved on to develop a PD concept tailored to the specific context of Alles Clara. Creativity and collaboration guided us in developing a concept for PD in Alles Clara—serving as the compass on our journey (see Figure 2). Defining our destination meant exploring counsellors' shared values and vision. Identifying key concepts for PD in Alles Clara led us to take first steps to bring the concept into action. Drawing on counsellors' experiences and perspectives, we shaped our approach to PD and jointly mapped the pathway towards sustaining a person-centred culture in online counselling. The quotes presented below are illustrative examples of perspectives that emerged repeatedly in workshop discussions.

#### 3.2.1 Shared values and vision

The shared values and vision of the counsellors demonstrate what they perceive as high-quality, person-centred counselling in Alles Clara. Counsellors agree upon that family caregivers need to feel heard, understood, taken seriously, safe and seen as individuals, as a person, in digital counselling, aligning with the underlying programme theory [Gabl et al., under review; (9)]. One counsellor expressed this sentiment: “Someone is listening to me—someone with professional expertise who understands the work involved and what needs to be done. […] Someone truly gets me. Nothing is sugar coated; my reality is acknowledged as it is—neither downplayed nor exaggerated. And that feels good to me” (WS1). To achieve this, counsellors work together with family caregivers to build relationships and remain authentic. “Because if you're not authentic, you can't provide good counselling” (WS2). They make decisions collaboratively with family caregivers while being fully present. “This ‘being there’ for and listening […] And this ‘being present’. We also discussed that this is what defines Alles Clara” (WS1). Counsellors give family caregivers the time they need. “I actually got the feedback: you can sense that—that someone is there, someone who takes their time for me” (WS1). In counselling, family caregivers receive a “space where they can be, be themselves, be at” (WS1). These accounts illustrate a collectively shared commitment: at its core, counselling in Alles Clara needs to be person-centred. “That's what quality is about—that we, as counsellors, turn to the person seeking advice” (WS1).

#### 3.2.2 Key concepts guiding practice development: knowledge, time and attitude

Drawing on counsellors' experiences and perspectives, we identified three key concepts for PD: Knowledge, time and attitude are needed to create a workplace where counsellors can flourish.

Counsellors' knowledge is considered essential for high-quality person-centred counselling in Alles Clara. Knowledge includes in-depth professional expertise and extensive practical experience, as well as research skills and the ability to find creative solutions to problems. Additionally, digital literacy, along with strong reading and writing skills, are crucial for text-based counselling. Counsellors bring “a great deal of interest and a desire for continuous development” (WS2). They share their knowledge within the team and benefit from mutual exchange. Encouraging their creativity and curiosity is key to fostering both their professional and personal growth, allowing them to continuously expand their expertise, their knowledge.

Another key to person-centred counselling lies in the time available to counsellors, time they can dedicate to their work and time they can give to family caregivers. Across workshops, counsellors repeatedly emphasized the importance of having sufficient time for both counselling and reflection. “For me, this is the ideal world: not having to act quickly or come up with an instant solution. It's ideal to have time, to not have to do something hastily just to calm a crisis” (WS2). Counsellors take the time needed for the person seeking advice, for finding solutions, and for conducting thorough research. To ensure person-centred counselling, they also have time to reflect on their work and engage in targeted learning and skill development, enabling them to continuously grow both professionally and personally.

A person-centred culture is characterized by a shared attitude within the counsellor team. Within their field of work, counsellors are recognized as experts and seen as individuals, as a person. They themselves are given space, time, and support to learn, develop, and grow [Gabl et al., under review; (9)]. Counsellors see themselves as part of Alles Clara and acknowledge their responsibility in realizing the vision of person-centred counselling for family caregivers seeking advice. “What it takes is passion, fire, courage, and conviction” (WS2). Person-centred counselling means placing the person—the family caregiver—at the center. Counsellors focus on the person they are engaging with, their unique situation, often going “beyond what I've ever done before […] that's when it truly matters to me” (WS2).

#### 3.2.3 From concept to action: implementing and testing tools for practice development

Shared values and vision and key concepts were mapped on the PD concept. To get this concept into action, to foster development on a personal level, supporting counsellors on their journey of individual transformation, authentic engagement and guided active learning are considered key strategies. Counsellors additionally defined basic principles which need to be adhered to in tools and measures for PD in Alles Clara: reflection, inspiration and participation.

Reflection includes both self-reflection—“to get a sense of myself” (WS2)—and reflection with others. “Sometimes you need to hear yourself think” (WS2). Group reflection allows counsellors to gain insight into their peers' counselling approaches and, in doing so, “reflect on their own practice—How am I actually doing?—by engaging in a positive and constructive comparison with others” (WS2). Counsellors find inspiration in rethinking their own counselling approaches, viewing challenging situations from a different angle and exploring new ways to problem solving. They benefit from sharing experiences with one another, “when something went well, when a particular phrase really resonated, when someone was brave” (WS2) and get inspired to refine their textual and linguistic expression. “There are always many ways” (WS2) to address family caregivers' concerns—taking inspiration from these different approaches allows counsellors “to flourish” (WS2), “to grow” (WS2), both personally and professionally. Creating space for counsellors' growth was perceived as essential for strengthening counselling practice in Alles Clara. Their active participation thus emerged as another fundamental principle. The concept of PD in Alles Clara must be something that “is allowed to grow with them […] something that develops” (WS2). To get the concept into action, we launched four mini-projects, each serving as a steppingstone toward a sustainable implementation.

Counsellors (1) identified and shared person-centred moments in counselling, deepening their reflection and strengthening their practice. They (2) enriched the online counselling handbook, transforming it into a dynamic resource tailored to their evolving needs. The facilitator (3) guided the first group reflection, fostering collective learning and professional growth. The development panel became a driving force for PD, with counsellors (4) actively shaping and evaluating the mini-projects. All four mini-projects were implemented successfully. Counsellors perceived the testing phase as “a valuable experience” (C10). The collaborative work within the team was described as particularly beneficial for “learning something new” (C02) and being able to “break new ground” together (C10). Across feedback formats, counsellors repeatedly emphasized that openness to new ideas and a respectful and open interaction with each other is considered key to creating a “productive work atmosphere” in which they can “grow together as a team, learn from each other, and […] expand competencies” (counsellor protocol).

“As a Development Panel, we were able to recognize ourselves as a team that goes through a shared process and has a common task to fulfil. As a group, we consistently had positive feelings of: cohesion, one stepping in for another, we are a team” (counsellor protocol).

### 3.3 Packing for the journey ahead: practice development strategies in Alles Clara

**Figure 3:**
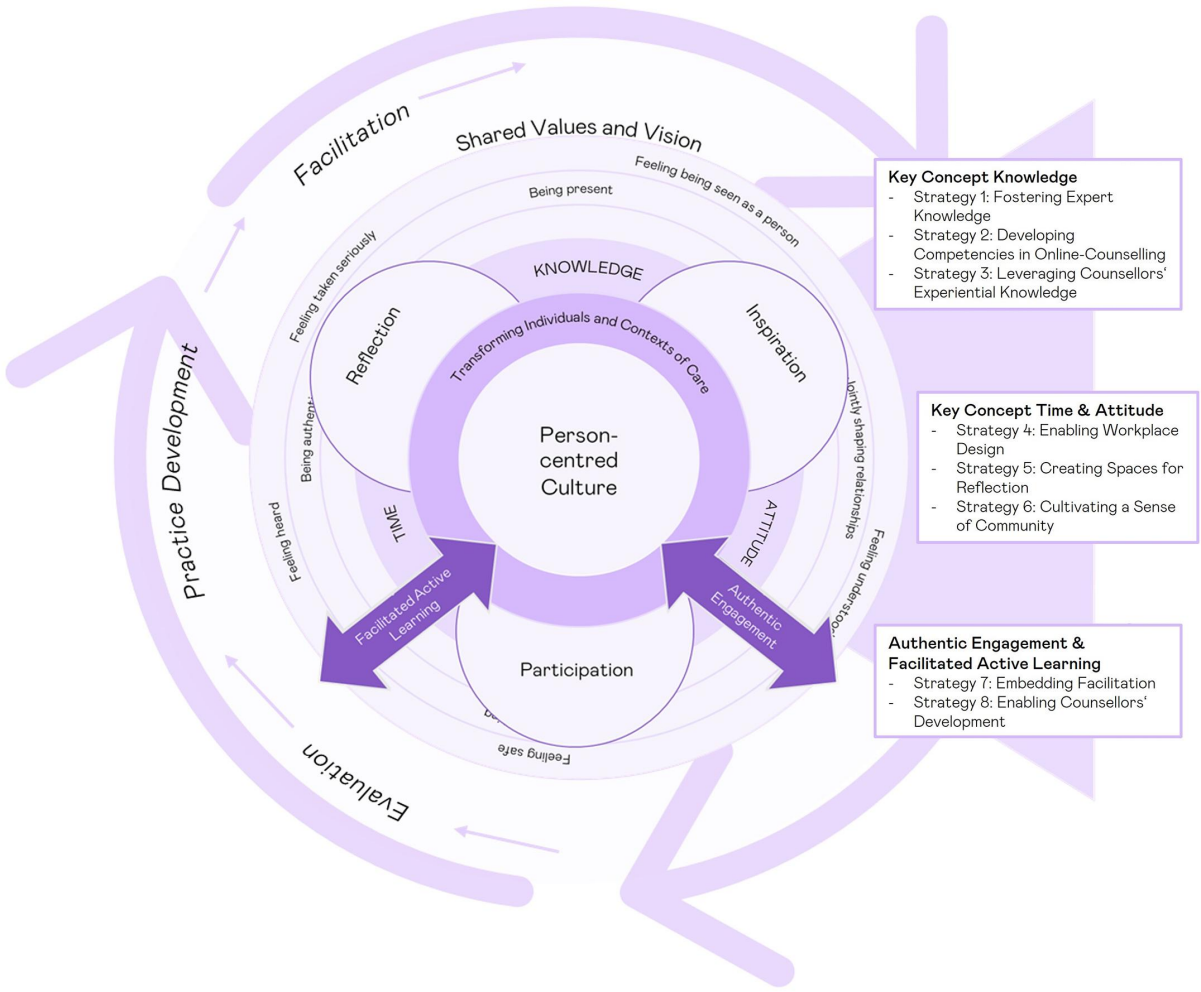
Practice Development Strategies in Alles Clara.

Over the course of the project, we identified and refined key measures and practical resources that have proven essential for supporting counsellors and strengthening their practice. (1) Knowledge, (2) Time & Attitude, and (3) Authentic engagement & guided active learning form the overarching structure for organizing eight key PD strategies in Alles Clara (see Figure 3). These strategies translate abstract ideals into practice-oriented objectives, delineate central mechanisms for achieving them, and structure established instruments and measures that support their realization. Empirical insights drawn from counsellors' experiences provide evidence of their relevance and effectiveness. The illustrative quotes presented illustrate recurring and collectively discussed perspectives. Taken together, the strategies offer a coherent framework for strategically guiding and sustaining PD in Alles Clara.

#### 3.3.1 Key concept knowledge: strategies for practice development

Charting the path to counsellors' knowledge entails pursuing three interconnected strategies. As a key concept shaping person-centred counselling, knowledge is not a static resource, but an evolving body guiding practice.

***Strategy 1: Fostering expert knowledge*** strengthens the team's collective expertise through professional exchange, structured knowledge management, and targeted training. The strategy aims to support ongoing professional development, establish a shared knowledge base, and systematically leverage counsellors' regional and specialized expertise. One key measure to support these objectives involves making the team's existing competencies and professional backgrounds visible. As one participant described, the team is “very diverse, individualists, all of whom have already had long working lives” (C01). This visibility not only reinforces counsellors' self-perception as experts but also facilitates access to specialised knowledge within the team: “More people know more than one alone” (C01). Another important measure is the facilitation of professional exchange among counsellors. This is enabled through a dedicated channel on the internal communication platform, transparent access to counsellors' contact details and working hours, and the provision of an openly accessible virtual meeting space. One participant highlighted the added value of this exchange: “You write into the counsellors' chat, and you get a response. And you get a response that actually helps you” (C05). In addition, shared knowledge is built through the ongoing expansion and collective curation of a central knowledge database. Counsellors continuously collect counselling-relevant content, which is stored on the internal communication platform. Continuous training represents another key measure for strengthening expert knowledge within the Alles Clara team. In 2024, for example, a workshop on digital ethics and an information session on legal guardianship for adults were offered. Counsellors emphasised the learning gains resulting from these collective efforts: “I think that as a group we have already come very far, because we communicate so much with each other, also on a professional level. I have learned a great deal” (C05). This perception of knowledge as a shared and collective resource emerged repeatedly across counsellors' accounts.

“This support is such an enrichment. When I started at Alles Clara, I felt very alone. Now, you always have access, you have phone numbers, you can call. Care is an incredibly broad field, and there is so much knowledge that no one person could ever cover alone. We are all experts in our own way, and I really appreciate that” (C05).

***Strategy 2: Developing competencies in online counselling*** focuses on equipping counsellors with the competencies required for effective and person-centred digital practice. A central measure is the initial training programme, which prepares counsellors for their work at Alles Clara. The training covers text-based online counselling, professional relationship building, process design, data protection, audio and video counselling, and crisis intervention in digital settings. To support practice-based learning, training and feedback sessions are conducted, both with members of the Alles Clara team and experienced counsellors. Another key resource is the online counselling handbook, developed specifically for Alles Clara as a practice-oriented guide. Counsellors actively contribute to its ongoing development, for example by creating text modules. As one counsellor notes, “I used these text modules extensively at the beginning! They were a big help” (C05). Even experienced counsellors continue to rely on the handbook: “I still look things up in my first handbook […] and I will continue to do so” (C10). Additional technical support is provided through manuals, short instructional videos, and a dedicated troubleshooting chat on the internal communication platform. “You can ask anyone for help at any time” (C05). Taken together, these measures strengthen counsellors' competencies, enhance their capacity for process design, further develop digital skills, and improve writing and reading proficiency.

“One client once wrote to me that my appreciative tone helped her enormously. The communication is incredibly empathetic. I learned a great deal. My text modules were literally lifesaving at the beginning. I always thought I could write fairly well—but this was on another level. It was really valuable” (C05).

***Strategy 3: Leveraging counsellors' experiential knowledge*** aims to enable systematic learning from professional experience and to initiate targeted development processes based on these insights. To this end, Alles Clara implemented a mentoring programme in which new counsellors are paired with experienced colleagues. Mentors share their experiences, answer questions, and support newcomers during their transition into the role. Counsellors highly value this exchange, and some mentoring relationships evolve into ongoing peer consultation groups: “Whenever I talk with my colleagues, I learn something new” (C05). Beyond individual mentoring relationships, the internal communication platform again plays a central role in organisational learning. Counsellors share experiences, perspectives, and suggestions through dedicated channels, complemented by regular feedback meetings. These measures support the systematic integration of practitioner knowledge into organisational development.

“And it's not just that we are counsellors—we also take on a kind of teaching role. We are allowed to pass things on to those who come after us, and that expands the counsellor role. That is something truly special” (C10).

#### 3.3.2 Key concepts time and attitude: strategies for practice development

Rooted in the key concepts of time and attitude, three strategies foster continuous development.

***Strategy 4: Enabling workplace design*** encompasses measures that allow counsellors to actively shape both their digital and physical work environments. Counsellors emphasize the importance of meaningful participation in design processes: “That someone asks us, that's not a given […] And it's extremely important that this exists and that we have this opportunity” (C02). Measures include user testing and feedback sessions aimed at the targeted development of the Alles Clara app. Regarding physical working conditions, flexibility and individualized solutions are emphasized, with the aim of promoting a supportive work environment and enhancing job satisfaction. Counsellors experience their needs as being recognised and respected, perceiving opportunities for workplace design as a sign of appreciation.

***Strategy 5: Creating spaces for reflection*** represents another central pillar and seeks to strengthen counsellors' reflexivity, deepen awareness of professional practice, and foster personal and professional development. As one counsellor reflects, “I reflect much more now than I used to. Before, I just did things without much reflection” (C06). A key measure in this context is supervision. Online supervision sessions are offered at regular intervals, with counsellors able to register independently based on individual needs; on average, participation occurs once per quarter. Group reflection is perceived as “a good preventive tool, helping us get along well, talk things through, and articulate what needs to be said” (C03). Additional reflective tools include a shared space for collecting and discussing person-centred moments in counselling, which encourages self-observation and learning—“that I observe myself a bit more and get to know myself better” (C05). Counsellors also complete self-assessment questionnaires following counselling sessions, while clients are invited to provide direct feedback. Both forms of feedback serve as important triggers for reflection. “We are allowed to reflect, and we have time to reflect” (C01).

***Strategy 6: Cultivating a sense of community*** aims to ensure open communication, strengthen a sense of belonging, and foster counsellors' shared commitment. As one counsellor explains, “When a group understands itself as a group and works that way, you develop a different kind of cohesion and sense of belonging” (C03). Measures include monthly counsellor meetings, community events, transparent communication, the use of multiple communication channels, regular updates, and the collective celebration of successes. “Because everyone works alone, initiatives like these are what actually create a team” (C06). Counsellors repeatedly emphasize the importance of the Alles Clara spirit and team culture: “You see yourselves as a unit, almost like a fabric you weave together, something that supports and holds you” (C03). The collaboration within the mini-projects further strengthened this sense of belonging and demonstrated “how a team can grow together even when you are not physically together” (C01). Community building is therefore regarded as a key strategy for PD in Alles Clara.

“What we will certainly take with us is the group dynamic and the sense of community. That is also the overarching message of Alles Clara. Being part of a community that strives for growth and development together is incredibly valuable” (C03).

#### 3.3.3 Authentic engagement & guided active learning: strategies for practice development

Authentic engagement and guided active learning are regarded as essential drivers of person-centred practice. Through the mini-projects and beyond, Alles Clara creates opportunities for counsellors to actively develop themselves, their professional practice, and the organisation as a whole.

***Strategy 7: Embedding facilitation*** aims to establish facilitation as a connecting element between individual counselling practice, collective team development, and organizational learning. A key insight emerging throughout the project was that facilitation was experienced as most valuable when embedded within existing relationships, shared experiences, and everyday collaboration, rather than introduced as an additional external element. Facilitation in Alles Clara is therefore to be understood as a practice-oriented, relational process that builds on existing structures, shared experiences, and established relationships within the team. Its purpose is to create spaces for collective learning, reflection, and creative exchange that directly relate to counsellors' everyday practice and contribute to the sustainable development of counselling practice in the digital realm. Measures include the implementation of creative formats, fostering collaboration, continuous reflection, and the joint exploration of practice-relevant topics. The facilitator's role was described as providing structure and coherence: “At first, I didn't even know what facilitation was, but it is necessary […] It brought all the ideas together in an important way” (C03). At the same time, counsellors emphasized the importance of professional closeness, familiarity, and trust for meaningful engagement within the digital setting, which increasingly shaped the understanding of facilitation as an embedded and relational practice. By embedding facilitation within everyday practice and aligning it closely with Alles Clara's mission and counsellors' shared vision, facilitation becomes a key strategy for supporting both counsellors' professional and personal growth.

***Strategy 8: Enabling counsellors' development*** aims to strengthen participation, further develop competencies, foster creativity and curiosity, and enable targeted improvements. PD “cannot just happen in an hour and a half one afternoon but happens continuously. That's exactly what it is” (C04). Key measures include involving counsellors in co-creation processes, providing tools and guidance, accompanying implementation, facilitating collective reflection, and celebrating successes. Counsellors have long been involved in development initiatives in Alles Clara, including the creation of the online magazine “Clartext” and the organization of informational events for family caregivers. Participation in the present project was described as “a truly valuable experience, because you were challenged again […] but that's what makes it special: you don't just do the same thing over and over; you get to explore new ground” (C10). Active involvement also strengthened counsellors' confidence in the meaningfulness of their work. Collaboration once again emerged as a central factor. Joint work was described as “team building, you really grow together as the Alles Clara team” (C06) and as “refreshing and nourishing” (C03). Strengthening the sense of community was identified as “a crucial secret” (C03). When asked what they would wish for in terms of PD, one counsellor expressed the following wish: “Please let's keep going, but in a different way. The worst thing is when someone tells you, ‘just stay the way you are’—that's nonsense. Instead, let's continue to develop together. That constant collective call to keep evolving is what matters” (C05). Through diverse formats, close collaboration within the team, and continuous reflection processes, Alles Clara's practice is shaped collaboratively with counsellors.

“What I learned was to look beyond my own boundaries. I hope this continues […] Projects like this are incredibly important. You learn, and you can pass something on to others. Everyone contributed; no one did nothing. That's what it's about—bringing people along, even those who might hesitate at first. So thank you for letting me be part of projects like this” (C10).

## 4 Discussion

This study set out to examine the potential of PD as an approach for fostering a person-centred culture within the digital context of online counselling for family caregivers.

Family caregivers constitute a central yet often overlooked pillar of contemporary care systems. Globally, they provide the majority of care for people living with chronic illness, disability, or frailty, contributing between 70% and 90% of all care work, often alongside employment and other responsibilities (4, 20, 21). Demographic ageing, increasing prevalence of chronic conditions, and changing family structures further intensify both the demand for family care and the complexity of caregiving roles (4, 22). While informal caregiving can be experienced as meaningful and enriching when adequate support is available (23, 24), a substantial proportion of family caregivers report high levels of burden and negative health consequences (5, 6). Digital services hold considerable promise for delivering accessible forms of support (25–27). Despite the growing number of digital services, however, systematic evidence of their impact remains thin (28, 29). A realist-informed evaluation of Alles Clara sought to close this gap. The scientific groundwork clearly underscores the significance of person-centredness in this context. Beyond mere rhetoric, the central mechanism, the crucial aspect driving Alles Clara's impact, lies in “the person-centred approach to counselling” [(3); Gabl et al., under review; (9)]. Anchored in the underlying programme theory, we moved beyond the conceptual debate on whether person-centred approaches align with digital care, instead focusing on the process of fostering person-centredness within the new and challenging context through PD.

Our journey unfolded in three key stages. First, mapping the landscape of counselling in Alles Clara established a contextual foundation for the road ahead. The design of the app influences whether and how family caregivers engage with the service. However, the true impact of the digital offering stems from the way counselling is structured and delivered [(3); Gabl et al., under review; (9)]. While the Realist evaluation provided a comprehensive assessment of the landscape, evolving needs require continuous re-evaluation of the specific context shaping counselling practice. We conducted a quantitative analysis of both the characteristics of counselling and the individuals seeking support, providing a first glimpse of our starting point. The results reinforce the previous findings [Gabl et al., under review; (9)]. Alles Clara is used by a diverse group, with counselling sessions characterized by heterogeneity. Post-counselling feedback revealed generally positive perceptions of the service, while simultaneously highlighting the complexity of counsellors' work and the need for a broad repertoire of professional skills and knowledge. Central to this complexity is the integration of knowledge-based support with emotional and relational accompaniment. In contrast to many existing digital support services for family caregivers, which predominantly focus on information provision, organization, coordination, or service navigation (25, 26), counselling in Alles Clara addresses the emotional and psychosocial dimensions of caregiving as well. In doing so, it foregrounds caregivers not merely as recipients of advice or information, but as persons embedded in complex personal, relational, and social contexts.

After mapping out the landscape we are navigating, we set out to explore the translation of PD into the digital realm. Because PD fundamentally relies on participation, this principle shaped our research approach as well. Rather than defining concepts in advance, we engaged in co-conceptualization with our research partners, the counsellors, to ensure that their insights and lived experience informed every step of the way. A culture of change can only emerge from within practice through active involvement of those who enact it. The CIP principles (collaboration, inclusion, and participation) (30) served us as a methodological compass, ensuring shared ownership and collective learning throughout the project. Consistent with prior research, co-creation proved transformative when counsellors were enabled to meaningfully shape their practice and experienced their contributions as consequential, fostering a collective commitment to the ongoing development of practice (31). Realising this transformative potential required consciously created spaces for dialogue and co-creation, embedded within cultural and temporal conditions that enabled sustained engagement, reflection, and collective learning (31, 32). This way, what began as an exploration into largely uncharted territory evolved into a transformative journey, undertaken collaboratively with counsellors, demonstrating that PD can foster person-centredness—even in an environment, where traditional cues of presence and connection are profoundly altered.

While Garbett and McCormack's PD framework (13) provided essential orientation, translating person-centred principles into digital practice is not a straightforward process. Rather, it requires careful adaptation to a context that strongly influences the way in which relationships are shaped—“in care practices and […] in the social world” [(33), p.63]. Developing a shared vision and agreeing on shared values emerged as vital: they guided the PD process and simultaneously served as a reflective compass for counsellors in shaping their practice. Exploring counsellors' vision of high-quality online counselling led us back to where we came from. Counselling in Alles Clara is about unique encounters, deeply personal interactions shaped by a person-centred approach, where experiences matter, and the success of a counselling session is individually defined. Counsellors' shared values and vision—offering family caregivers a space to be, be themselves, be at—be seen as a person—align with the underlying programme theory [Gabl et al., under review; (9)]. While “working together to build relationships”, “remaining authentic”, “making decisions collaboratively” and “being fully present”, aligning with the fundamental principles of person-centred practice (34), counsellors commit to going the extra mile to offer meaningful support.

Yet the two core driving mechanisms of PD, authentic engagement and facilitated active learning, emerged as the most significant points of tension in Alles Clara's digital environment. Authentic engagement requires human connection and sympathetic presence. Facilitated active learning depends on shared reflection and dynamic interaction (35). These processes must be intentionally redesigned to work across the digital interface, within the ongoing tension between “being seen” and “not being seen” (3). The adapted PD framework provided a conceptually grounded structure for organizing context-sensitive strategies and measures, translating abstract ideals into tangible, actionable practices within a digital environment. Our eight strategies constitute a coherent framework for guiding and sustaining PD in Alles Clara. While many of the underlying measures are already part of everyday counselling practice, their consolidation into overarching strategies enables more intentional use, targeted adaptation, and continuous development within a complex digital work context. Strategies structured around knowledge focus on fostering expertise, developing competencies in online counselling, and systematically drawing on counsellors' experiences, with exchange and collective learning as central mechanisms. Time and attitude are addressed through strategies that support enabling working conditions, structured reflection, and a strong sense of community. These dimensions are closely interrelated and mutually reinforcing, and PD emerges through their combined enactment rather than through isolated interventions. Authentic engagement and guided active learning underpin strategies that foreground facilitation and counsellor empowerment. Participation cuts across all strategies, anchoring PD as a collective, ongoing process shaped with and by counsellors. Embedding facilitation emerged as another key strategy for PD in Alles Clara, which, however, had not previously been part of the counselling practice.

The first steps toward implementing facilitation were taken during the project by introducing a dedicated facilitator and conducting an initial group reflection, which revealed considerable potential. Nevertheless, the lessons learned throughout the project led us to presume that a sustainable implementation would require further testing and careful adaptation to the specific context of Alles Clara. Following the initial implementation, facilitation underwent further investigation in 2025 with the aim of anchoring it within Alles Clara as an effective and enduring PD strategy. Within this process, it became apparent that the work of the dedicated facilitator was fraught with challenges and had difficulty meeting expectations (36). Implementation was conceptualised as an evolutionary and progressive process of professional development and expertise building rather than a linear roll out (37). However, introducing an additional external element into a virtual workspace marked by physical distance tended to reinforce experiences of detachment and unfamiliarity, rather than fostering engagement, connection and trust. In response to these observations, facilitation in Alles Clara was fundamentally re-positioned. Rather than being conceived as an additional or stand-alone role, it was reframed as an integrated, relational practice embedded within Alles Clara's existing structures and everyday practice (36). This shift proved highly valuable and directly informed the refinement of Strategy 7: “Embedding facilitation”. By anchoring facilitation in established relationships, professional closeness, and the lived realities of counsellors, facilitation was able to become a vehicle for connection and “being seen” and in doing so, contributing to the establishment of a person-centred culture (36).

These experiences underscore a key insight from translating PD into the digital realm. Strategies developed for analogue care contexts cannot be transferred unchanged but must be carefully reworked in response to digitally mediated conditions. In Alles Clara, authentic engagement is strengthened through deliberate efforts to cultivate relational closeness despite physical distance. Purposefully created opportunities for connection, such as in-person community days, complemented by a low-threshold digital space for everyday exchange, proved essential for sustaining relationships and a shared sense of belonging among counsellors. Building on existing relationships and peer-based reflection proved more effective in facilitating active learning than introducing external hierarchies or rigid structures. Creating spaces of closeness and mutual support enables authentic exchange, strengthens collective learning, and forms the foundation of a sustainable person-centred culture.

For PD to succeed in digital healthcare, maintaining human connection must remain a deliberate and continuous effort. Digital technologies fundamentally reshape rather than merely supplement care relationships, actively influencing how connections are formed, experienced and maintained (33). This resonates with recent work emphasising that person-centred care in digital settings depends on deliberately embedding the person's presence, story, and relational experience into digital environments, rather than allowing efficiency or functionality to dominate practice (38). As healthcare systems embed technology-based interventions more comprehensively, the stakes of getting this balance right intensify. While technology has always been present in healthcare, its systematic integration represents a paradigm shift in how care is conceptualized, organized, and experienced. Success hinges not solely on technological sophistication, but critically on the professionals who deploy it, the organizational cultures that contextualize its use, and the broader socio-political environment shaping care delivery (39). PD provides a coherent and context-sensitive approach to navigating this paradigm shift, as it simultaneously supports practitioners' reflexivity and flourishing while drawing attention to the organisational and infrastructural conditions that enable trust, connection, and learning (40). In doing so, PD offers a viable pathway for aligning technological advancement with the growing call for a humanistic framing of healthcare, ensuring that digital transformation strengthens rather than erodes the relational core of care.

## 5 Limitations

This study explored whether and how PD can support counsellors in Alles Clara to provide person-centred digital counselling. As such, the findings are shaped by the specific context of a single digital counselling service, characterized by its organisational culture, value base, and setting. The PD framework and strategies were co-created to address the unique needs, structures, and constraints of Alles Clara. While this limits direct transferability to other digital care services, the work provides principles, processes, and methodological insights that may inform PD in comparable contexts if adapted to local conditions. Participation in the development panel was voluntary, which may have resulted in selection bias, as counsellors with a stronger interest in PD or organisational development may have been more likely to engage in the project and related reflection processes.

Second, while the study provides rich insights into practitioner experience, organisational learning, and the structuring of person-centred PD, it does not directly assess outcomes for family caregivers. Although the study builds on prior realist evaluation findings identifying person-centredness as the service's central mechanism, conclusions regarding the impact of PD on service user experiences remain indirect and should be interpreted cautiously. Although PD is theoretically associated with the development of person-centred and high-quality care, the present study did not assess whether the identified PD strategies resulted in measurable improvements in counselling quality and family caregiver outcomes. Accordingly, any assumptions regarding improvements in counselling quality should be understood as theoretically and empirically informed propositions rather than directly demonstrated outcomes of the present study. Future research should therefore investigate more directly whether and how PD influences service user experiences and outcomes in digital counselling contexts.

Third, the close collaboration between researchers and practitioners, which formed a central methodological feature of the participatory design, may also have introduced social desirability bias within workshops, reflections, and feedback processes. Participants may have been more inclined to emphasize positive experiences or align with shared project goals. Similarly, the researchers' prior involvement with Alles Clara and commitment to person-centred practice may have shaped data interpretation and analytical emphasis. To address this, reflexive discussions were conducted continuously within the research process. In addition, findings were developed collaboratively through ongoing dialogue among researchers and counsellors to support critical reflection and ensure that interpretations remained grounded in participants' experiences.

Finally, digital (counselling) contexts are dynamic and continuously evolving. The strategies and facilitation approaches developed in this study reflect the technological and organisational configuration of Alles Clara at the time of implementation. Ongoing adaptation will be required as digital infrastructures, modes of interaction, and organisational conditions change, reinforcing the view of PD in digital care as an ongoing, iterative process rather than a fixed model.

## 6 Conclusion

Ongoing debates around digitalisation in healthcare often position digital technologies as either humanising or dehumanising forces. Our findings suggest that this dichotomy is misleading. Digitalisation does not, in itself, determine whether care becomes more or less person-centred; rather, this depends on how practitioners interpret, enact, and sustain person-centred values within digitally mediated contexts. Person-centred digital counselling does not emerge from tools, but from the beliefs, attitudes, and everyday actions of those providing care. Understanding the digital realm as the context in which care unfolds shifts attention away from technology itself and towards professional stance and practice. PD can play a critical role in supporting this shift by enabling practitioners to reflect on, articulate, and align their beliefs and practices with person-centred principles.

As digital environments increasingly influence care delivery, sustaining person-centred practice at the micro-level depends on coherent support from meso- and macro-level conditions. While digital contexts offer clear benefits, they also pose risks to connection, trust, and relational closeness. Our findings underscore the need to intentionally design organisational and infrastructural conditions that respond to practitioners' needs, enabling trustful relationships and a strong human core to flourish. In Alles Clara, this is reflected in opportunities for real-life encounters, structured spaces for engagement and reflection, and accessible communication channels that actively counteracted distance and support authentic engagement. The adapted PD framework provides a coherent structure for organizing a set of developmental strategies and measures, translating abstract values into tangible, actionable practices within a digital environment. For PD to succeed in digital healthcare, maintaining human connection must remain a deliberate and continuous effort.

Digitalization in healthcare is neither optional nor reversible; it is an ongoing transformation of how care is organized, delivered and experienced. The key question, therefore, is not whether to digitalise care, but how person-centred practice can be preserved and sustained within digital contexts. In this sense, packing for the journey ahead becomes a necessity: equipping ourselves with the principles, processes, and attitudes that preserve the human core of care, even as digital transformation continues to reshape practice. PD offers a compass for navigating this terrain, ensuring that the person remains at the very centre of care.

## Data Availability

The raw data supporting the conclusions of this article will be made available by the authors, without undue reservation.
